# Relationship Between Industry Funding and Otologists

**DOI:** 10.1002/ohn.1331

**Published:** 2025-06-04

**Authors:** Natalia M. Hajnas, Annik S. Lupieri, Angelica M. Mangahas, Leah Butler, Heather M. Weinreich

**Affiliations:** ^1^ Department of Otolaryngology–Head and Neck Surgery University of Illinois at Chicago Chicago Illinois USA; ^2^ University of Illinois College of Medicine Rockford Rockford Illinois USA; ^3^ Department of Obstetrics and Gynecology University of Illinois at Chicago Chicago Illinois USA

**Keywords:** consulting, female surgeons, funding opportunities, gender bias, gender disparities, healthcare industry, industry funding, mentorship, surgery, transparency

## Abstract

**Objective:**

To determine whether allocation of industry funding to otologists/neurotologists differs by gender.

**Study Design:**

Cross‐sectional retrospective analysis.

**Setting:**

Online open‐access database.

**Methods:**

Non‐research payments made to otologists/neurotologists in 2019 and 2020 included in the Centers for Medicare and Medicaid Services Open Payments Database were examined. Comparison analyses were performed after removing outliers.

**Results:**

In 2019 and 2020, 217 otologists received industry funding. Funding was in the form of consulting, charity, education, gifts, grants, honoraria, royalty/license, travel and lodging, and other compensation. Only 15.7% were women. After controlling for age and years in practice, of the total sum of payments, women only received 4.4% (*P* < .001). Of the total number of payments, women only received 12.5% of payments (*P* = .07). Men received funding within each category, whereas women did not receive funding in honoraria, other compensation, royalty, gift, or charity. Within education, women received a median of 6 payments and men received 1 (*P* = .01). With travel and lodging, the median payment per male physician was $1013 ($581.96‐$1785.71) versus $684.26 ($274.03‐$1078.5) per female physician (*P* = .002).

**Conclusion:**

Gender disparities in industry funding exist amongst otologists/neurotologists. Women receive less money as a whole and are underrepresented in several payment categories. Even after controlling for age and years in practice, women continue to receive less money. Therefore, these gender disparities that exist are not explained by age or experience.

The collaboration between physicians and the healthcare industry has become an integral part of modern medical practice.^1^ Although industry funding can support research, education, and technological advancements through user input, it may also raise concerns related to conflicts of interest and potential gender disparities in funding and opportunities.^2^, ^3^ Industry funding for surgeons comes in various forms, such as research grants, speaker honoraria, sponsorship of conferences, consulting fees, gifts, and support for medical education.^3^

Although the number of females entering the medical profession and surgical specialties is increasing, disparities in industry relationships based on gender continue to exist.^4^ Academic physicians tend to receive more industry funding compared to nonacademic physicians.^5^ Multiple studies have indicated that females comprise a smaller proportion of academic physicians, particularly in vascular surgery, urology, plastic surgery, radiation oncology, and otolaryngology.^5^, ^6^, ^7^, ^8^, ^9^, ^10^, ^11^ Therefore, this may affect their access to industry funding opportunities.

Overall, female surgeons have fewer relationships with industry and receive less industry payments compared to male surgeons.^7^, ^10^, ^12^, ^13^, ^14^, ^15^, ^16^, ^17^, ^18^ Weiss et al^12^ evaluated industry funding across multiple specialties. The study examined the percentage of funding given to female physicians compared to the percentage of females in the given subspeciality and found that 14 out of 17 specialties showed a smaller proportion of female surgeons receiving industry funding, including otolaryngology. Studies consistently indicate that the average payment for female surgeons is significantly less than payments to male surgeons.^12^, ^14^ This remains true for all categories of payment; however, Weiss et al^12^ found that female physicians received more funding for research compared to male physicians, specifically female colorectal surgeons, general surgeons, surgical oncologists, and pediatric surgeons. Yet, other studies still indicate that male urologists and plastic surgeons receive more research funding compared to their female counterparts.^9^, ^11^

Increased industry payments were also associated with increased academic rank.^10^ Male physicians were more likely to be chairs, presidents, deans, division chief professors, professors, and associate professors, whereas female physicians were more likely to be assistant professors.^6^, ^8^, ^10^, ^11^, ^19^ Despite this, when stratified by rank, a lower median payment for females was still demonstrated compared to males at the same academic rank.^6^

In 2021, of the 372 board‐certified neurotologists in the United States, only 13% were female, showing a gradual increase in diversity.^20^ Although representation of women in otolaryngology is improving, this has not translated to financial ties with industry.^21^ Female otolaryngologists are less likely to have industry relationships and received lower amounts of financial support when compared to their male counterparts.^22^ A study using the Open Payments Database in 2014 showed a total of $4,945,631 went to academic otolaryngologists that were included in the study. In total, 88.5% of that total sum was allocated to male otolaryngologists.^22^ This study also evaluated median payments while controlling for academic rank and found that male otolaryngologists still had a higher median payment compared to female otolaryngologists. Males also received a greater proportion of total funding compared to the gender composition of each subspecialty among all subspecialties and had higher median contribution levels among otologists, laryngologists, and rhinologists.^22^ This study, however, only evaluated a population of academic otolaryngologists and reported summarized results.

Although studies have indicated that female otolaryngologists receive less industry funding opportunities and smaller average payments, there is no study that examines otologists/neurotologists specifically. Additionally, there is no existing study that analyzes disparities in industry funding as a function of age and career stage. Therefore, the objective of this study was to determine whether allocation of industry funding to otologists/neurotologists differs by gender while controlling for age and career stage.

## Methods

This study was a cross‐sectional retrospective analysis of a large open‐access database. The population included in this study was otologists and neurotologists. Data were obtained from the publicly available Centers for Medicare and Medicaid Services (CMS) Open Payments Database. Reporting entities, such as drug and medical companies, input information regarding financial relationships with physicians in the CMS database.^23^ Physicians then have a 2‐month period during which they may volunteer to review and dispute certain data. After those 2 months, the data are published. All payments must be reported, with the exception of payments less than a specific threshold, adjusted each year. During the years 2019 and 2020, these thresholds were USD 10.79 and USD 10.97, respectively. The data are publicly available and met the exemption from review by the institutional review board at the University of Illinois at Chicago (protocol #2019‐0270). Industry funding from January 1, 2019, to December 31, 2020, in the form of non‐research payments was included and combined for analysis. Data collection occurred during May 2022 to June 2022, and data for the year 2021 were not yet available. Non‐research payments include travel and lodging, consulting, honoraria, education, other compensation, royalty, non‐research grants, gifts, and charity.

The following information was obtained on each individual during May 2022 to June 2022: gender, age, subspecialty type (otology/neurotology), and board completion year. Gender and age were obtained from U.S. News & World Report's Doctor Finder, Healthgrades, Doximity or the individuals' academic or private practice website. These websites were searched in the above‐stated order such that an individual's name was entered into the search function on U.S. News & World Report's Doctor Finder, and gender and age were recorded. If either of these variables were not listed on this website, each subsequent website was searched. The CMS data set does include the subspecialty type, but this was confirmed or modified based on the American Board of Otolaryngology–Head and Neck Surgery public database (ABOHNS), Doximity, or the individuals' academic or private practice website. The subspecialty was only changed or modified according to the academic or private practice website if the fellowship was specific and with a trackable institution provided. The year each individual completed their board exam was obtained from the ABOHNS public database.

Comparison analyses were performed after removing outliers using Tukey methodology, which is based on the interquartile range (IQR). Far‐out values, defined as Q1 – 3 × IQR and Q3 + 3 × IQR were removed from each gender both by total payments and by total sum.

Percentages were calculated to determine the rate within dichotomous variables. For normally distributed continuous variables (age), means were calculated. For nonnormally distributed variables (board completion year), medians with IQR were calculated. *t* test was used to compare normal distributed variables (age), a Mann‐Whitney *U* test was performed to compare nonnormally distributed variables (payments per physician), and a chi‐square test for categorical variables (total sum, total payments). Board completion year was used as a surrogate marker for career stage.

A Fisher's exact test was conducted to compare the proportion of each gender receiving funding across different categories. Multiple regression analysis was used to determine the relationship between total industry sums and payments and age, career stage and gender. A *P*‐value of <.05 was considered significant. Where appropriate, 95% confidence intervals were calculated. All statistical analyses were carried out using MedCalc Statistical Software version 22.016 (MedCalc Software).

## Results

In total, 217 otologists received industry payments. Of these, 15.7% (n = 34) were women and 84.3% (n = 183) were men. From the total sample population collected for otologists/neurotologists receiving industry funding between January 1, 2019, and December 31, 2020, 5 female and 16 male outliers were identified and removed. In total, 196 otologists were analyzed after outliers were removed. There were 29 women (14.8%) and 167 men (85.2%). Age was obtained for 72.4% (n = 21) women and 80.8% (n = 135) men. For age, women were significantly younger than men (49 vs 54.1, mean difference 5 years, *P* = .02). Career stage information was obtained in 89.7% (n = 26) women and 92.8% (n = 155) men. Women were more likely to complete boards later than men as the board median year was 2009 (IQR 2005‐2014) for women and 2003 (IQR 1994‐2011) for men, indicating an earlier career stage.

**Figure 1:**
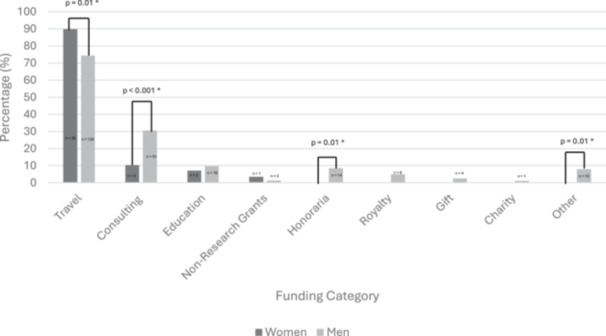
Proportion of men and women who received funding by category. *Statistically significant at *P* < .05.

For all surgeons, the total sum of industry funding was $508,777. As a population, men received 96% ($486,277) of the total sum, whereas women received 4% ($22,500). The total number of payments (or transactions) was 771. Men received 88% (n = 675) and women received 12% (n = 96) of payments. Men received funding within each category, whereas women did not receive funding in honoraria, other compensation, royalty, gift, or charity (Figure 1).

**Figure 2:**
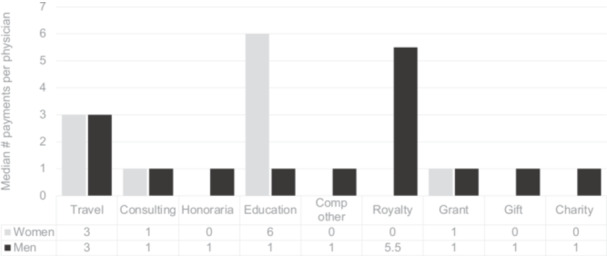
Number of payments. This is a comparison of the median number of payments per physician by category.

Figure 2 compares the median number of payments per physician by category. Within education, women received more payments, with a median of 6 payments, whereas men received 1 (*P* = .01).

**Figure 3:**
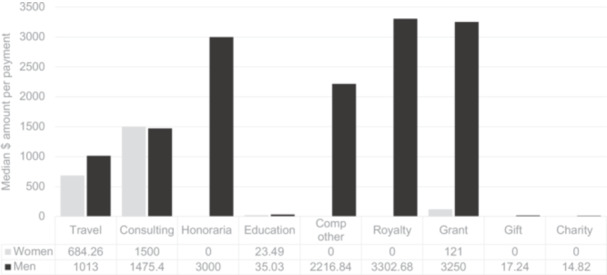
Dollar amount per payment. This is a comparison of the median dollar amount per payment per physician by category.

Figure 3 compares median dollar amount per payment per physician by category. Within travel and lodging, the median payment per male physician was $1013 (IQR $582.0‐$1,785.7) versus $684.3 (IQR $274.0‐$1078.5) per female physician (*P* = .002). The grant category also shows a higher dollar amount per male physician; however, it could not be statistically compared as the n was 1 for women in this category.

A multiple regression was run to assess correlations between the independent variables of age, gender, and year of board examination and the dependent variable “total payments received.” The regression results are summarized in Table 1. The results indicate that holding all other variables constant (ie, age and career stage), being female was correlated with 1.7 fewer payments as compared to men (*P* = .04). Interestingly, for every increase in age by 1 year, holding all other independent variables constant, a decrease in total payments received by 0.27 was observed.

**Table 1:** Multiple Regression for Total Number of Payments

Regression results: total number of payments			
Variables	Coefficients	Standard errors	*P* values
Constant	415.1		
Gender	−1.7	0.82	.04*
Age	−0.27	0.10	.008*
Year completed boards	−0.2	0.09	.03*
*R* ^2^			
Adjusted *R* ^2^		0.06	
*F* ratio			
Ratio		4.27	
Significance level		*P* = .006	

* Statistically significant at *P* < .05.

A second regression was run to assess correlations between the independent variables of age, gender, and year of board examination and the dependent “total sum” awarded in US dollars by industry. The regression results are summarized in Table 2. The results show that holding all other variables constant (ie, age and career stage), being female was correlated with a statistically significant decrease in total sum awarded by $2928.1 when compared to men (*P* = .006). The only statistically significant independent variable was found to be gender. Age and career stage were not statistically significantly correlated with the total sum of money given to otologists/neurotologists.

**Table 2:** Multiple Regression for Total Sum of Payments

Regression results: total sum (in US dollars)			
Variables	Coefficients	Standard errors	*P* values
Constant	−27,633.5		
Gender	−2928.1	835.6	.0006*
Age	−30.1	103.7	.77
Year completed boards	16.3	94.83	.86
*R* ^2^			
Adjusted *R* ^2^		0.06	
*F* ratio			
Ratio		4.4	
Significance level		*P* = .005	

* Statistically significant at *P* < .05.

## Discussion

The study revealed a persistent gender disparity in industry funding amongst otologists/neurotologists that is not explained by age or career stage. Despite comprising 15% of the study population, women received only 4% of the total amount of money distributed by industry. Women also received a smaller number of payments from industry (12%); however, this was more comparable to the population distribution. The number of payments was also influenced by age and career stage, thus not exclusively related to gender. This gender disparity is particularly evident in specific payment categories, with women notably absent from honoraria, other compensation, royalty, gift, or charity payments. Men, on the other hand, were represented in each payment category. Although 30% of men received consulting opportunities, only 10% of women did. Additionally, differences in payment amounts were observed, with women receiving less money for travel and lodging compared to their male counterparts. Although the grant category also suggested a higher dollar amount in men, the statistical comparison is limited due to the small sample size of women in this category. Within the education category, women received a significantly higher median number of payments compared to men. These differences in funding distribution raise questions about the factors contributing to these disparities.

The regression analyses provided several interesting data points. Most notably, being male is correlated with a higher amount of money provided by industry, both when looking at the total sum of payments in US dollars and the number of total payments made. Furthermore, age as a variable appeared to be negatively correlated with the total number of payments. This was an unexpected result, showing that as age increases, one should expect fewer total payments according to the model utilized. A 0.27 difference is likely not clinically meaningful. If considered relevant, this may be a product of a more complex relationship between age and industry funding that is not captured in the regression model. It may also be that as the number of payments decreases, the sum amount paid increases. Age was not found to be statistically correlated with the sum amount of dollars given by industry. This phenomenon may be due to small sample sizes, a nonlinear correlation between age and amount of funding, a collinear relationship between age and another variable, or lastly because of an unknown variable that is not captured by this data set.

Evaluating the root cause of gender differences can be postulated, though the scope of this study was primarily to present these differences. One hypothesis is that implicit biases and gender‐related barriers within the field may influence funding allocation. These barriers may include a lack of opportunity, not being approached, or not being in the same professional circle that gets notice of these opportunities. Alternatively, it is also possible that women have different priorities or interests when it comes to relationships with industry. Prior discussions have suggested that perhaps women do not pursue or value industry relationships as much and that women may perceive a greater conflict of interest or have more distrust in industry relationships.^14^ Although these latter postulations could be true, these would only explain the underrepresentation of women in general, but not necessarily the disparities in transaction amounts. Our data support the notion of possible implicit biases, as women received more payments for education than men, whereas men dominated in categories like consulting. These findings suggest a nuanced landscape where gender‐related factors may impact the opportunities and financial support available to otologists.

Gender disparities in industry payments are not unique to otology/neurotology. Similar patterns have been reported in other medical specialties, pointing towards systemic issues within healthcare.^7^, ^10^, ^12^, ^13^, ^14^, ^15^, ^16^, ^17^, ^18^ Within otolaryngology, Eloy et al^22^ were the first to delve into breaking down these differences and showing disparities. Our study contributes to filling the gap in understanding these disparities within otology specifically and looks at a more recent time point (2019‐2020 vs 2014).

It is important to acknowledge the limitations of our study. Our study primarily relies on the accuracy of the CMS Open Payments Database for the correct reporting of industry contributions. Though these data may be subject to biased self‐reporting as individuals can dispute and correct data that are attributed to themselves, it provides a large database unlike any other discussing financial relationships between otologists/neurotologists and industry. A further limitation in our study is the small female sample size in several payment categories, which limits the robustness of statistical comparisons between men and women in those categories. Additionally, certain data collected, such as gender, are reliant on the accuracy of external websites including the U.S. News & World Report's Doctor Finder, which may not be based on self‐reported data. Furthermore, the setting of work is not described in this study, and there may be differences in industry funding based on academic versus private practice appointments. Of note, the data examined in this study spanned the years 2019 and 2020, which include the COVID‐19 pandemic and may have shown skewed results due to a disproportionate burden of the pandemic felt by women in the workplace.^24^, ^25^ Lastly, as mentioned previously, we cannot conclude the root cause of the differences observed. Our study's strength lies in its comprehensive analysis of industry payments in a specific group, otology, with consideration of age and career stage as well as examining not only total sums but also the number of interactions (transactions) with industry.

There are many advantages and disadvantages of working in the industry. Disadvantages include potential conflicts of interest, whereas advantages include the impact on instrument design, usability, and insight into industry research. They allow networking and help establish physician consultants as key opinion leaders. Relationships with industry and industry‐supported financial contributions can impact instrument design and usability, research endeavors, educational opportunities, access to innovative products, and scholarly advancement.^5^, ^10^

Future research should evaluate additional predictors of industry funding trends and investigate top earners as they may provide insight into successful and robust industry relationships. Research‐specific payments should be explored as well. Industry type and composition of industry stakeholders may provide another interesting take on the patterns seen here. Finally, future research should delve deeper into the root causes of these disparities, exploring the impact of networking opportunities, biases of the industry stakeholders, and women's opinions and prioritization of industry relationships. Ultimately, these efforts are vital for ensuring equal opportunities for all otologists/neurotologists, irrespective of gender, thus promoting more equitable opportunity and compensation.

## Conclusion

Our data have revealed that gender disparities in industry funding exist amongst otologists/neurotologists after controlling for age and career stage. Women receive less money as a whole and within fewer payment categories. There are several potential advantages in industry relationships, and we should aim to provide the same level of opportunity and compensation for men and women.

## Author Contributions

**Natalia M. Hajnas**, design, data acquisition, data interpretation, drafting, revising, presentation of the research, approval of final manuscript; **Annik S. Lupieri**, data acquisition, drafting, revising, approval of final manuscript; **Angelica M. Mangahas**, data acquisition, drafting, revising, approval of final manuscript; **Leah Butler**, data acquisition, approval of final manuscript; **Heather M. Weinreich**, conception, design, data interpretation, revising, approval of final manuscript.

## Disclosures

### Competing interests

Heather M. Weinreich reports a relationship with Stryker that includes employment. Heather M. Weinreich is currently an employee of Stryker Corporation. At the time this work was completed, she was not employed by Stryker.

### Funding source

No funding was provided by Stryker in support of this research.
